# AFYA BORA CONSORTIUM FELLOWSHIP: a journey of success in Global Health Leadership Training

**DOI:** 10.4314/ahs.v21i1.1S

**Published:** 2021-05

**Authors:** Onesmus Gachuno, Theresa Odero, Esther Seloilwe, David Urassa, Edith Tarimo, Damalie Nakanjako, Nelson Sewankambo, Ndeso S Atanga, Edie G Halle-Ekane, Yukari Manabe, Kristin N Hosey, Susan A Chapman, Douglas J Wiebe, Joachim Voss, Gabrielle O'Malley, Yohana Mashalla, Margaret Ndegwa, Jarim Omogi, Carey Farquhar, Judith N Wasserheit

**Affiliations:** 1 University of Nairobi; 2 Working group; 3 University of Botswana; 4 Muhimbili University of Health & Allied sciences; 5 Makerere University; 6 University of Buea, Cameroon; 7 John Hopkins University; 8 University of Pennyslyvania; 9 University of Washington; 10 Afya Bora M&E

Epidemics and disease outbreaks often overwhelm existing health care systems and infrastructure, making them ill prepared to respond to these crises. In the 1990s, the health infrastructure of many African countries in Sub-Saharan Africa (SSA) was overwhelmed by the HIV/AIDS pandemic. In 2007, the World Health Organization (WHO) recommended six core components or “building blocks” that included leadership and governance as essential ingredients of a functional health system that could efficiently deliver equitable services.^1^ Many countries in SSA, together with their European, American or Asian development partners, put great efforts into strengthening and aligning these health systems building blocks to improve the health of their citizens and stem the tide of the HIV pandemic. Major efforts were called for to improve HIV prevention and care in tandem with the Millennium Development Goals, prominently including the 90-90-90 UNAIDS targets.^2^ To accelerate progress on these goals and targets, and to enhance effectiveness of health systems in SSA, there has been a call for a new transformative approach to health worker training to include leadership training beyond the usual technical skills training.^3^,^4^

To contribute to bridging the gap of transformative leadership in health care, the Afya Bora Consortium Fellowship (ABC) was developed to train health care leaders for global health at ministries of health, academic institutions as well as governmental and non-governmental organizations to promote evidence- based health care and public health services in SSA.^5^ After a series of virtual planning meetings, a stakeholders meeting was held in Nairobi in 2009 to structure the fellowship program and make the training responsive to the training needs. The initial members of the consortium included faculty from the Schools of Medicine, Public Health, and Nursing from the Universities of Botswana, Nairobi in Kenya, Makerere in Uganda, Muhimbili in Tanzania, the University of Washington in Seattle, John Hopkins University, the University of California San Francisco, and the University of Pennsylvania. Later, the program was scaled up to include the University of Bueya in Cameroon and leading Chinese universities supported by the China Medical Board. The program is led by a team consisting of two members per institution; one with medical and one with nursing background. These Working Group members hold weekly virtual meetings and bi-annual face-to-face meetings (midterm and end-of-fellowship) to guide, evaluate and iteratively improve implementation of the program.

Fellows participate in a highly interdisciplinary one year training that includes: week-long face-face modules on key skills needed by global health leaders, including Leadership, Communications and Media, Global Health Policy and Governance, Monitoring and Evaluation, Human Resource and Budget Management, Qualitative methods, Effective Grant Writing, Implementation Science and Health Systems Research. Fellows receive up to nine months of mentored practical leadership experience at a local institutions (Health Facility or NGO ) in the partner countries. In addition, during their experiential attachment period, fellows use distance learning modules to strengthen their understanding of Research & Analytic Methods, Responsible Conduct of Research and Practice, HIV as a Global Health Challenge, and Program Management.^6^

Each fellow is mentored by at least two people, a working group member and a mentor from the respective attachment site for the experiential placement. In each country, attachment sites were selected based on the strength and capacity to mentor and provide experiential training to fellows. Fellows undertake a project which may have a research or a practice focus. Fellows' progress is routinely documented by an established structured M&E framework.^7^

With the lessons learned from the COVID 19 pandemic experience, ABC is transitioning most of the modules to virtual training to allow fellows to continue learning despite international travel restrictions. The Fellowship has set up a strong alumni platform to encourage networking and long-term collaboration in research, training and leadership in global health.

Global health-focused authors have repeatedly called for inter-professional training in Global Health to enrich interdisciplinary perspectives and embed teamwork approaches to overcome healthcare challenges.^3^,^4^ In infusing inter-professional training attributes, ABC has, since its inception, trained together 187 fellows (98 nurses, 78 Medical Doctors and 11 public health-related professionals).^7^ According to a 2018 survey, all African graduates from the fellowship have returned to serve in their countries to provide leadership skills to strengthen health systems and improve health service during the COVID-19 pandemic and into the future.^8^ This supplement was informed by the need for some of the Alumni to tell their stories in the course of their training.
